# Synthesis and Characterization of Fe_0.8_Mn_0.2_Fe_2_O_4_ Ferrite Nanoparticle with High Saturation Magnetization via the Surfactant Assisted Co-Precipitation

**DOI:** 10.3390/nano11040876

**Published:** 2021-03-30

**Authors:** Kornkanok Rotjanasuworapong, Wanchai Lerdwijitjarud, Anuvat Sirivat

**Affiliations:** 1 Conductive and Electroactive Polymers Research Unit, The Petroleum and Petrochemical College, Chulalongkorn University, Bangkok 10330, Thailand; kornkanok.rotj@gmail.com; 2 Department of Materials Science and Engineering, Faculty of Engineering and Industrial Technology, Silpakorn University, Nakorn Pathom 73000, Thailand; lerdwijitjarud_w@su.ac.th

**Keywords:** manganese ferrite, surfactant-assisted co-precipitation, SDS surfactant concentration, Iron (II) substitution, soft ferromagnetic behavior

## Abstract

Manganese ferrite nanoparticles (MnFe_2_O_4_) were synthesized via surfactant-assisted co-precipitation, where sodium dodecyl sulfate (SDS) was used as the template to control particle size at various SDS concentrations. The substitutions of iron (II) (Fe^2+^) into the MnFe_2_O_4_ ferrite nanoparticles were carried out to obtain Fe_(1_–_x)_Mn_x_Fe_2_O_4_, with various Mn^2+^: Fe^2+^ molar ratios. The synthesized ferrite nanoparticles were characterized by the Fourier-transform infrared spectroscopy (FT-IR), thermogravimetric analyzer (TGA), X-ray diffractometer (XRD), energy dispersive X-ray (EDX), X-ray photoelectron spectroscopy (XPS), scanning electron microscope (SEM), transmission electron microscope (TEM), two-point probe, and vibrating sample magnetometer (VSM) techniques. The experimental Mn:Fe mole ratios of the Fe_(1−x)_Mn_x_Fe_2_O_4_ ferrite nanoparticles were verified to be in agreement with the theoretical values. The synthesized MnFe_2_O_4_ and Fe_(1−x)_Mn_x_Fe_2_O_4_ ferrite nanoparticles were of mixed spinel structures, with average spherical particle sizes between 17–22 nm, whereas the magnetite ferrite nanoparticles (Fe_3_O_4_) were of the inverse spinel structure. They showed soft ferromagnetic behavior. The synthesized Fe_0.8_Mn_0.2_Fe_2_O_4_ ferrite nanoparticle possessed the highest saturation magnetization of 88 emu/g relative to previously reported work to date.

## 1. Introduction

Magnetic transition oxides or spinel ferrite nanoparticles are among the magnetic nanoparticles widely used in industrial and biomedical applications such as drug delivery [1], drug release [2], removal of heavy metal from water [3], catalyst [4], permanent magnet [5], tissue engineering [6], hyperthermia [7,8,9,10], magnetic resonance imaging (MRI) [11], gas sensor [12], and actuator [13], due to their thermal, optical, electrical, and magnetic properties [14,15]. The general spinel ferrite formula is AB_2_O_4_, where A and B are metallic cations residing at two crystallographic sites: the tetrahedral (A site is a divalent ion such as Mn^2+^, Ni^2+^, Co^2+^, Fe^2+^, Cu^2+^, and Zn^2+^) and the octahedral (B site is a trivalent ion, usually is Fe^3+^) [16]. Among the spinel ferrite nanoparticles, manganese ferrite (MnFe_2_O_4_) is a mixed (partially inverse) spinel structure with good properties such as high anisotropy, high Curie temperature, size-dependent saturation magnetization, low coercivity, and superparamagnetic behavior at room temperature [17,18]. Nevertheless, the properties of the manganese ferrite nanoparticles depend on their particle sizes and shapes, which are controlled by various synthesis conditions: the type of salt used, the reaction temperature, the pH value, the stirring rate, the ionic strength of the media, the M (divalent ion) to Fe^3+^ ratio, and the synthesis method [19,20,21].

Many methods have been used to synthesize manganese ferrite nanoparticles, such as sol-gel [22], thermal decomposition [23], solvothermal [24], micro-emulsion [25], and co-precipitation [18]. Among the synthesis routes, co-precipitation is one preferred method due to its simplicity, low cost, high yield, ease to control particle size, processibility at lower temperature, and environmentally friendly synthesis route [18,26,27]. However, the magnetic nanoparticles as obtained from this method normally aggregate, thus altering magnetic properties. Therefore, the functionalization and modification, such as using metals, polymer coating, and surfactants absorption on the surface, have been found to reduce the aggregation [21,28,29]. Recently, a cobalt ferrite nanoparticle (CoFe_2_O_4_) was synthesized by the co-precipitation method at various cetyltrimethylammonium bromide (CTAB) concentrations. The aggregation decreased with increasing CTAB surfactant [30]. Furthermore, the substitution at the tetrahedral with different divalent cations led to the changes in the magnetic properties. Modaresi et al. synthesized Mn_x_Fe_3−x_O_4_ ferrite nanoparticles by the co-precipitation method, with x varying from 0 to 1. The saturation magnetization increased with increasing Mn^2+^ ion content, as the Mn^2+^ ion magnetic moment is higher than the Fe^2+^ ion [31]. Other works on the manganese ferrite synthesis were reported with the saturation magnetizations and particle sizes to be between 15–69 emu/g and 5–46 nm, respectively [9,17,18,23,24,32,33,34,35].

In this work, the synthesis of Fe_(1−x)_Mn_x_Fe_2_O_4_ ferrite nanoparticles was carried out via the surfactant-assisted co-precipitation method. The physical, thermal, electrical, magnetic, and dispersion properties of the synthesized Fe_(1−x)_Mn_x_Fe_2_O_4_ ferrite nanoparticles were investigated under the effects of the surfactant type, the SDS surfactant concentration, and the Mn^2+^: Fe^2+^ molar ratio substitution, where x’s value was varied from 1 to 0 (x = 1, 0.6, 0.2, and 0). It will be shown that SDS was successfully used as the template to control the particle size and hence the saturation magnetization; the highest saturation magnetization value to date of 88 emu/g was obtained with x equal to 0.2 and with the nanoparticle size of 18 ± 3 nm exhibiting soft ferromagnetic behavior.

## 2. Materials and Methods

### 2.1. Materials

Iron (III) chloride (FeCl_3_∙2H_2_O, 99%, Sigma-Aldrich, Munich, Germany), manganese (II) chloride (MnCl_2_, Synthesis grade, Merck, Darmstadt, Germany), iron (II) sulfate (FeSO_4_∙7H_2_O, 99%, Univar, Ajax Finechem Pty Ltd, Australia), hexadecyltrimethylammonium bromide (CTAB, ≥96%, Fluka, Fluka Chemiclas Limited, Dorset Gillingham, UK), sodium dodecyl sulfate (SDS, Omnipur, Merck, Darmstadt, Germany), polysorbate20 (Tween20, Sigma-Aldrich, Lyon, France), 25 wt% Ammonium hydroxide solution (NH_4_OH, AR grade, Merck, Darmstadt, Germany), deionized water, and ethanol (AR grade, RCI Labscan, Bangkok, Thailand) were obtained and used without further purification.

### 2.2. Synthesis of Ferrite Nanoparticles

The synthesis of ferrite nanoparticles via the surfactant-assisted co-precipitation method was carried out in two parts.

#### 2.2.1. Effect of Surfactant Types and Concentrations

The synthesis of MnFe_2_O_4_ ferrite nanoparticles was carried out at various SDS concentrations. First, 0.81 g of FeCl_3_∙2H_2_O was mixed with 0.32 g of MnCl_2_ at the Fe^3+^:Mn^2+^ molar ratio of 0.10:0.05 and then dissolved in 25 mL deionized water. The SDS solutions, at the 0.5, 1.0, and 1.2 CMC concentrations (CMC is the Critical Micelle Concentrations of surfactant), were separately prepared by dissolving 0.058 g, 0.12 g, and 0.15 g in 25 mL deionized water at room temperature. Then, the prepared metal precursor solution was mixed with SDS solution. The mixture solution was vigorously stirred at 150 rpm and room temperature of 27 °C for 1 h to obtain a homogeneous solution and then heated up to 80 °C under a nitrogen gas flow. Then, 25 wt% NH_4_OH solution (19 mL) was added into the mixture solution dropwise, in which an orange solution changed into a black solution. The mixture solution was vigorously stirred at 80 °C for 4 h for the precipitation to proceed. Finally, the black precipitate was obtained through cooling down to room temperature, centrifugation, washing several times with deionized water and ethanol to remove the residue surfactant, and drying in an oven at 80 °C for 24 h to obtain the MnFe_2_O_4_ ferrite nanoparticles. The MnFe_2_O_4_ ferrite nanoparticles with CTAB and Tween20 were prepared by the same procedure at the surfactant concentration of 1.0 CMC.

#### 2.2.2. Effect of Mn^2+^:Fe^2+^ Molar Ratios

**Scheme 1:**
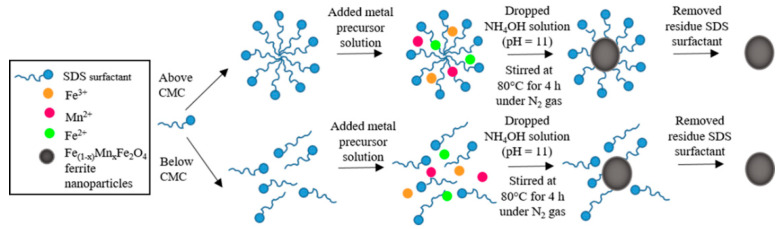
Schematic of Fe_(1−x)_Mn_x_Fe_2_O_4_ ferrite nanoparticle synthesis at various SDS surfactant concentrations.

The Fe_(1−x)_Mn_x_Fe_2_O_4_ ferrite nanoparticles were synthesized at various Mn^2+^: Fe^2+^ molar ratios, where x’s value was varied from 1 to 0 (x = 1, 0.6, 0.2, and 0). The Fe^3+^: Mn^2+^: Fe^2+^ molar ratios of the metal precursor solutions were 0.10:0.05:0.00 (0.81 g:0.32 g:0.0 g), 0.10:0.03:0.02 (0.81 g:0.19 g:0.28 g), 0.10:0.01:0.04 (0.81 g:0.063 g:0.56 g), and 0.10:0.00:0.05 (0.81 g:0.0 g:0.70 g); they were prepared under the same procedure as above but at the 1.2 SDS CMC, in which FeSO_4_∙7H_2_O was mixed with FeCl_3_∙2H_2_O and MnCl_2_ and then dissolved in 25 mL deionized water at room temperature. The bare MnFe_2_O_4_ ferrite nanoparticle was also prepared at the same condition without the surfactant to compare with the MnFe_2_O_4_ ferrite nanoparticles synthesized using SDS. The synthesized Fe_(1-x)_Mn_x_Fe_2_O_4_ ferrite nanoparticles were coded as Fe_(1−x)_Mn_x_Fe_2_O_4__yCMC_SDS, where x is the fractional molar ratio of Mn^2+^: Fe^2+^ and y is the fraction of the CMC of SDS. The synthesis schematic of the Fe_(1−x)_Mn_x_Fe_2_O_4_ ferrite nanoparticles at various SDS concentrations is shown in Scheme 1.

### 2.3. Characterization of Ferrite Nanoparticles

The structure of the MnFe_2_O_4_ ferrite nanoparticles was characterized for the functional groups by a Fourier transformed infraredspectrometer, FT-IR (Thermo Scientific, Nicolet iS5, Therno Fisher Scientific, Waltham, MA, USA) in the wavenumber range of 650 cm^−1^ to 4000 cm^−1^. Each sample was mixed with the ground KBr and then compressed.

A thermogravimetric analyzer, TGA (Perkin Elmer, TGA7, PerkinElmer, Waltham, MA, USA) was used to determine the thermal behavior and to confirm the structure of the MnFe_2_O_4_ ferrite nanoparticles. Each sample powder of 5–10 mg was loaded into a platinum pan as temperature was varied from 37 °C to 700 °C with the heating rate of 10 °C/min under a nitrogen flow.

A wide-angle X-ray diffractometer, XRD (Rigaku, SmartLab, Rigaku Corporation, Tokyo, Japan) was used to study the crystal structure of the ferrite nanoparticles. The CuK-alpha radiation source was operated at 40 kV/30 m and a K-beta filter was used to eliminate the interference peak. Each sample was dried, ground, compressed by a hydraulic machine to obtain a disc shape, and then placed on the sample holder. The 2θ range was from 10 deg. to 80 deg. with the scan speed of 2°/min and the scan step of 0.02°. 

A high-resolution field-emission scanning electron microscope with energy-dispersive X-ray, FE-SEM-EDX (Hitachi, S-4800 FE-SEM-EDX, Hitachi High-Tech Science Corporation, Tokyo, Japan) was used to determine the chemical composition ratios of the ferrite nanoparticles. Each sample was distributed on the sample holder and then coated with a thin platinum layer by ion-sputtering. The copper grid was used as the reference. 

An X-ray photoelectron spectroscopy, XPS (Kratos, Axis Ultra DLD, Manchester, UK) was used to examine the atomic percentages of Fe, Mn, and O atoms of the ferrite nanoparticles. The experiment was operated by a monochromatized A1 K source in the wide scan mode.

A high-resolution field-emission scanning electron microscope, FE-SEM (Hitachi, S-4800 FE-SEM, Hitachi High-Tech Science Corporation, Tokyo, Japan) was used to investigate the morphology and to measure the particle sizes of the ferrite nanoparticles. Each sample was distributed on a sample holder and then coated with a thin platinum layer by ion-sputtering. The SEM images were obtained by using an acceleration voltage of 2 kV, a current of 10 mA, and a magnification of 70 kx. The average particle sizes were determined from the SEM images from at least 100 random nanoparticles. 

A transmission electron microscope, TEM (Hitachi, H-7650, Hitachi High-Technologies Corporation, Tokyo, Japan) was also used to determine the particle shapes and sizes of the ferrite nanoparticles. Each sample was dispersed in ethanol, and the solution was coated onto a copper grid. The instrument was operated at 100 kV.

The electrical conductivity of the ferrite nanoparticles was measured by an electrometer (Keithley, 6517A, Tektronic, Portland, OR, USA) connected to a custom-built two-point probe. Each sample was ground and compressed by a hydraulic machine to obtain a disc shape. The applied voltage and the resultant current were plotted to obtain the slope, which was used to calculate the electrical conductivity of the ferrite nanoparticles by using Equation (1) [13]:

σ = 1/ρ = 1/(R_s_ × t) = I/(K × V × t) = slope/(K × t), (1)

where σ is the specific conductivity (S/cm), ρ is the specific resistivity (Ω cm), R_s_ is the sheet resistivity (Ω), I is the measured current (A), K is the geometric correction factor (1.00 × 10^−4^), V is the applied voltage, and t is the disc thickness (cm).

A vibrating sample magnetometer, VSM (LakeShore, Series 7400 model 7404, Lake Shore Cryotronics, Inc., Westerville, OH, USA) with a 4 in electromagnet was used to measure the magnetic properties of the ferrite nanoparticles, namely the saturation magnetization (M_s_), coercivity (H_c_), and remanent magnetization (M_r_). The hysteresis loops were measured under the magnetic field strength of 10,000 Gauss at room temperature. The data were taken at 80 points/loop with a scan speed of 10 s/point.

## 3. Results

### 3.1. Characterization of Ferrite Nanoparticles

In the surfactant assisted co-precipitation method of the ferrite nanoparticles, SDS was used as the surfactant template. The mechanism of co-precipitation reaction is represented by the following Equation (2) [36]:

2Fe^3+^ + 6OH^−^ 2Fe(OH)_3_

xMn^2+^ + 2OH^−^ xMn(OH)_2_

(1 − x)Fe^2+^ + 2OH^−^ (1 − x)Fe(OH)_2_

2Fe(OH)_3_ + xMn(OH)_2_ + (1 − x)Fe(OH)_2_ Fe_(1−x)_Mn_x_Fe_2_O_4_ + 4H_2_O (2)

In the synthesis mechanism, the base solution (NH_4_OH) was added into the mixed metal ion precursor solution (Fe^3+^, Mn^2+^, and Fe^2+^) to precipitate the ferrite nanoparticles; the OH^-^ from NH_4_OH interacted with the metal ion precursors to form the hydroxide precipitant and by-product water. The presence of SDS in the synthesis provided the micelles which stabilized the metal ion precursors by the interaction between the SO_4_^2−^ polar group of SDS and the metal cation precursors [37]. SDS interacted with the precipitant surface by the bridges of hydroxide precipitant; thus, SDS prevented the aggregation of ferrite particles at a nano-scale [38]. Finally, SDS was removed by washing with water and ethanol. The synthesis reaction under N_2_ atmosphere prevented the oxidation of Fe^2+^ ions to Fe^3+^ ions by the O_2_ atmosphere [21]. The substitution of MnFe_2_O_4_ ferrite nanoparticles with Fe^2+^ ions by varying the Mn^2+^: Fe^2+^ molar ratio was known to affect the magnetic properties [39]. 

#### 3.1.1. FT-IR Analysis

**Figure 1:**
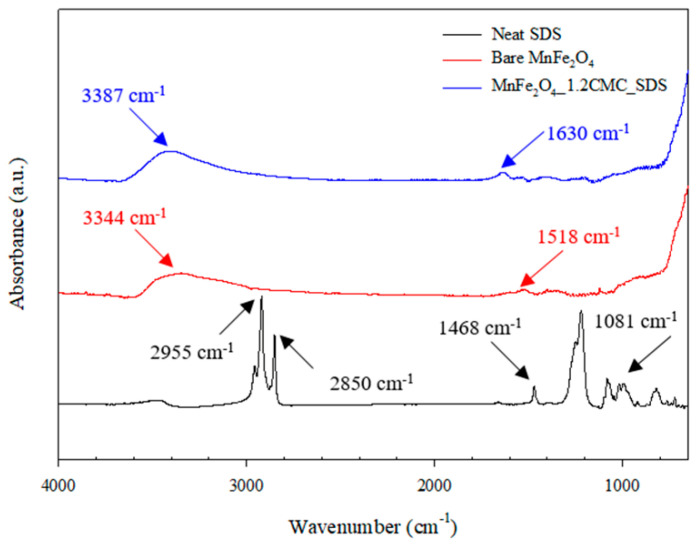
FT-IR spectra of the neat SDS surfactant, bare MnFe_2_O_4_, and MnFe_2_O_4__1.2CMC_SDS ferrite nanoparticles.

The chemical structure of the MnFe_2_O_4_ was investigated by FT-IR spectroscopy as shown in Figure 1. The FT-IR spectrum of SDS showed peaks at 1081 cm^−1^, corresponding to the S-O stretching vibration; 1468 cm^−1^, corresponding to the C-O stretching vibration; and 2955 and 2850 cm^−1^, corresponding to the C-H stretching vibration [37]. The FT-IR spectra of the bare MnFe_2_O_4_ and MnFe_2_O_4__1.2CMC_SDS show the peaks at 3344–3387 cm^−1^ and 1518–1630 cm^−1^, corresponding to the bending and stretching vibrations of the O-H groups, respectively. These peaks occurred from the humidity [31]. The FT-IR spectrum of MnFe_2_O_4__1.2CMC_SDS indicates that SDS was not present. Thus, it can be concluded that the SDS was completely eliminated by washing with water and ethanol.

#### 3.1.2. TGA Analysis

**Figure 2:**
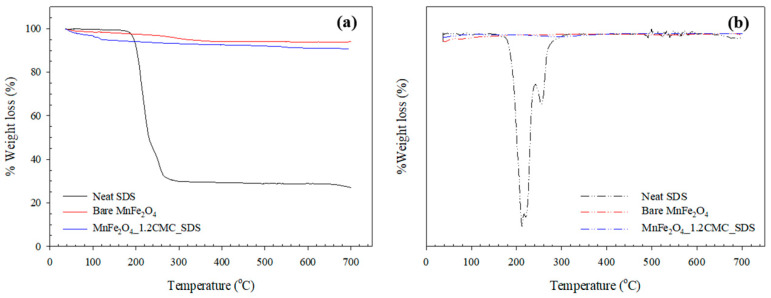
Thermograms of the neat SDS surfactant, bare MnFe_2_O_4_ and MnFe_2_O_4__1.2CMC_SDS ferrite nanoparticles: (**a**) TGA curve; and (**b**) DTG curve.

The thermograms (TGA) and derivative thermograms (DTG) of the MnFe_2_O_4_ are shown in Figure 2a,b. The SDS thermogram shows a significant weight loss between 180 and 350 °C of about 69.18%, due to the degradation of SDS [40]. The degradation temperature of the SDS surfactant is identified at 212.34 °C from the DTG curve in Figure 2b. The bare MnFe_2_O_4_ has a weight loss of 3.66% between 100 and 350 °C from the removal of adsorbed water, whereas the MnFe_2_O_4__1.2CMC_SDS has the weight loss of 4.79% between 60 and 160 °C due to the water evaporation [41]. The thermograms suggest that the ferrite nanoparticles consist of over 95% inorganic phase of the MnFe_2_O_4_, and the synthesized MnFe_2_O_4_ is of good thermal stability. Furthermore, the MnFe_2_O_4__1.2CMC_SDS has no weight loss related to the SDS surfactant. This confirms that the SDS surfactant was totally removed from the MnFe_2_O_4__1.2CMC_SDS.

#### 3.1.3. X-Ray Diffraction (XRD) Analysis

Generally, the spinel ferrite has a structural formula of AB_2_O_4_ (where A and B are the divalent and trivalent metallic cations in the tetrahedral and octahedral sites, respectively) with the spinel ferrite crystal structure. For the MnFe_2_O_4_ ferrite, it is a face-centered cubic mixed spinel structure (Fd3m) with a structural formula of (Mn^2+^_1−c_Fe^3+^_c_)_A_[Mn^2+^_c_Fe^3+^_2−c_]_B_O_4_. The substitution of divalent iron ions (Fe^2+^) changes the occupancy of the mixed (partially inverse) spinel in the MnFe_2_O_4_ crystal to the inverse spinel crystal structure of Fe_3_O_4_ crystal with the structural formula of (Fe^3+^)_A_[Fe^2+^Fe^3+^]_B_O_4_ [27,42,43]. 

**Figure 3:**
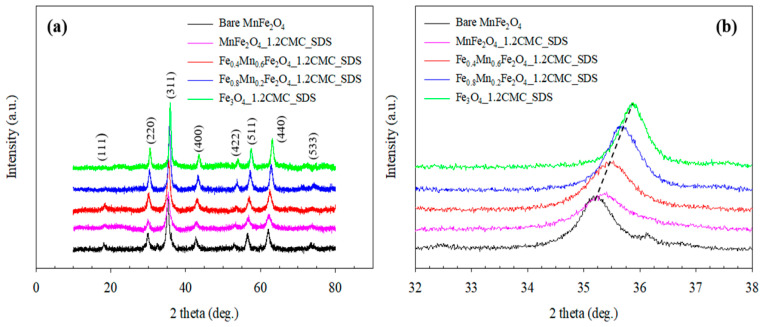
XRD patterns of: (**a**) the synthesized bare MnFe_2_O_4_ and Fe_(1-x)_Mn_x_Fe_2_O_4__1.2CMC_SDS ferrite nanoparticles; and (**b**) the peak position of (311) diffracted as a function of molar ratios of Mn^2+^:Fe^2+^.

The XRD patterns of the bare MnFe_2_O_4_ and Fe_(1-x)_Mn_x_Fe_2_O_4__1.2CMC_SDS, with various Mn^2+^: Fe^2+^ molar ratios, where x’s value varies from 1 to 0 (x = 1, 0.6, 0.2, and 0), are shown in Figure 3a. The XRD patterns of the Fe_(1−x)_Mn_x_Fe_2_O_4__1.2CMC_SDS at various Mn^2+^:Fe^2+^ molar ratios show the same XRD patterns as the bare MnFe_2_O_4_ with the diffraction peaks at the (111), (220), (311), (400), (422), (511), (440), and (533) crystal planes indicating the cubic spinel structure with the Fd3m space group [27,31,39,44]. Furthermore, each pattern does not show the diffraction peaks of other components, which may occur from the oxidation reaction. This confirms that the metal ion precursors (Fe^3+^, Mn^2+^, and Fe^2+^) were completely bounded to the ferrite nanoparticles [27].

All of the magnetic ferrite XRD patterns show the main diffraction peak at around 2θ ≈ 35.1°–35.9° corresponding to the (311) crystal plane, and the averaged crystalline size (D) was calculated from this peak by using the Debye-Scherrer’s Equation (3) [45]:

D = Kλ/(βcosθ)(3)

where D is the average crystallite size (nm), K is the shape factor that depends on the structure of the crystallite (in this case, the crystallite shape is spherical; K = 0.9), λ is the X-ray wavelength (Cu Kα-radiation; 0.154056 nm), β is the full width at half-maximum (FWHM) of the main diffraction peak (331) (radian), and θ is the Bragg’s angle (radian). The averaged crystallite sizes are tabulated in Table 1. From Table 1, the averaged crystallite size of the MnFe_2_O_4_ ferrite nanoparticles at various SDS concentrations decreases with increasing SDS concentration from 10.6 nm to 7.80 nm. This is because the surfactant influences the particle–particle interaction at the nano-scale; the higher surfactant concentration corresponds to the weaker particle–particle interaction, and the crystallite size is reduced [30]. The averaged crystallite size of Fe_(1−x)_Mn_x_Fe_2_O_4__1.2CMC_SDS increases with increasing Fe^2+^ cation substitution (with x = 1 to 0) from 7.80 nm to 13.3 nm, as the increase in Fe^2+^ ions substitution increases the crystal growth rate, leading to the crystallite size enlargement [46]. Moreover, the (311) crystal plane peak is shifted toward the higher angle with increasing Fe^2+^ ions, and the broadened peaks imply smaller crystallite sizes, as shown in Figure 3b [47]. The lattice constant (a) was calculated from the (311) crystal plane by using Equation (4) [14];

(4) $$a\;=\;d\sqrt{h^{2}+k^{2}+l^{2}}$$

where d is the interplane distance, and (hkl) are the Miller indices of the crystal planes. The volume of unit cell (V_cell_) was calculated by using Equation (5) [45];

V_cell_ = a^3^ (5)

The lattice constants and the volumes of the unit cell are listed in Table 1; these two parameters decrease with increasing SDS surfactant concentration, and the Fe^2+^ ions substitution (x). The decrease of the lattice constant with increasing Fe^2+^ ions substitution (x) is due to the ionic radius effect. The larger ionic radius of Mn^2+^ (0.83 Å) in the ferrite nanoparticles structure is replaced by the smaller ionic radius of Fe^2+^ (0.78 Å), resulting in a decrease in the lattice constant and consequently the volume of unit cell [48]. The hopping length for tetrahedral site (L_A_) and octahedral site (L_B_) were calculated by Equations (6) and (7), respectively [49]:

(6) $$L_{A}\;=\;a\sqrt{3}/4$$

(7) $$LB_{\;}=\;a\sqrt{2}/4$$

The hopping length L_A_ and L_B_ values are tabulated in Table 1. The hopping lengths L_A_ and L_B_ under the effect of SDS concentrations change insignificantly, while the hopping lengths L_A_ and L_B_ under the effect of increasing Fe^2+^ ions substitution (x) decrease slightly. The L_A_ and L_B_ values are related to the lattice constant values as shown in Table 1. The X-ray density ($\rho_{\mathrm{x-ray}}$) and the specific surface area of nanoparticles are calculated by Equations (8) and (9), respectively [50]:

(8) $$\rho_{\mathrm{x-ray}}\;=\;8M/N_{A}a^{3}$$

S = 6/Dρ_x-ray_ (9)

where M is the molecular weight of the sample (g/mol), N_A_ is Avogadro’s number (6.02 × 10^23^ mol^−1^), and D is the crystallite size (nm). The X-ray density values and specific surface areas are tabulated in Table 1. The specific surface area of Fe_(1−x)_Mn_x_Fe_2_O_4__1.2CMC_SDS decreases with increasing Fe^2+^ ions content, as the surface area is inversely related to the particle size that increases. A larger particle size corresponds to a smaller specific surface area and vice versa [31]. From the XRD results, the differences in crystallite size, lattice constant, the volume of unit cell, hopping length of L_A_, hopping length of L_B_, and specific surface area confirm that the Fe^2+^ ions were successfully substituted into the MnFe_2_O_4_, consistent with previous works [31,39,41,50].

#### 3.1.4. EDX and XPS Measurements 

**Figure 4:**
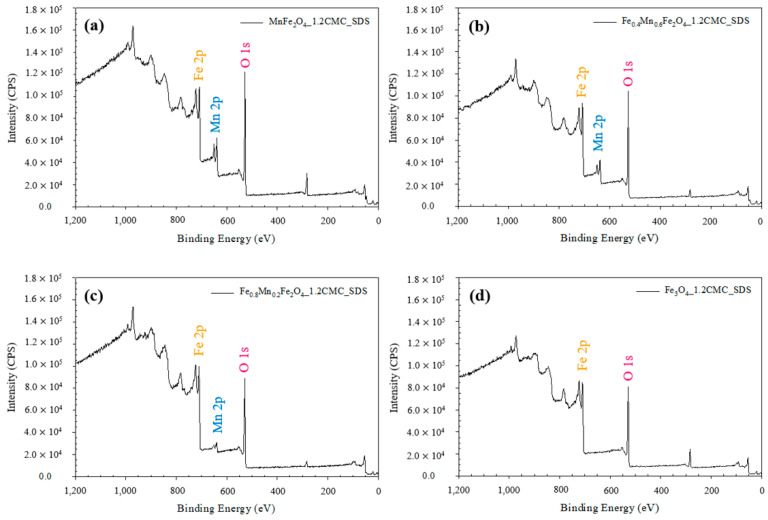
XPS spectra of the synthesized Fe_(1-x)_Mn_x_Fe_2_O_4__1.2CMC_SDS ferrite nanoparticles: (**a**) MnFe_2_O_4__1.2CMC_SDS, (**b**) Fe_0.4_Mn_0.6_Fe_2_O_4__1.2CMC_SDS, (**c**) Fe_0.8_Mn_0.2_Fe_2_O_4__1.2CMC_SDS, and (**d**) Fe_3_O_4__1.2CMC_SDS.

The chemical compositions of Mn:Fe in each Fe_(1−x)_Mn_x_Fe_2_O_4__1.2CMC_SDS were verified by the atomic percentages of Fe, Mn, and O atoms from the EDX and XPS measurements. From the EDX results, the Mn:Fe molar ratios of MnFe_2_O_4_, Fe_0.4_Mn_0.6_Fe_2_O_4_, and Fe_0.8_Mn_0.2_Fe_2_O_4_ were 1:2.02, 1:3.82, and 1:13.64, respectively. These results indicate that the Fe element increases with an increasing amount of Fe^2+^ ions substitution in the MnFe_2_O_4_, and the experimental Mn:Fe ratios were quite close to the theoretical molar ratios, namely 1:2, 1:4, and 1:14, respectively. The survey scan XPS spectra of the ferrite nanoparticles are displayed in Figure 4. Each XPS spectrum illustrates the binding energy peaks of 706.1 eV, 650.1 eV, and 529.2 eV, corresponding to the Fe 2p, Mn 2p, and O 1s, respectively [51]. The Mn:Fe molar ratios for the MnFe_2_O_4_, Fe_0.4_Mn_0.6_Fe_2_O_4_, and Fe_0.8_Mn_0.2_Fe_2_O_4_ from the XPS results are 1:2.02, 1:4.09, and 1:14.03, respectively. These values are close to the EDX results and the theoretical molar ratios. Hence, the EDX and XPS results confirm that the Fe^2+^ ions were successfully substituted into the MnFe_2_O_4_.

#### 3.1.5. SEM and TEM Analysis

**Figure 5:**
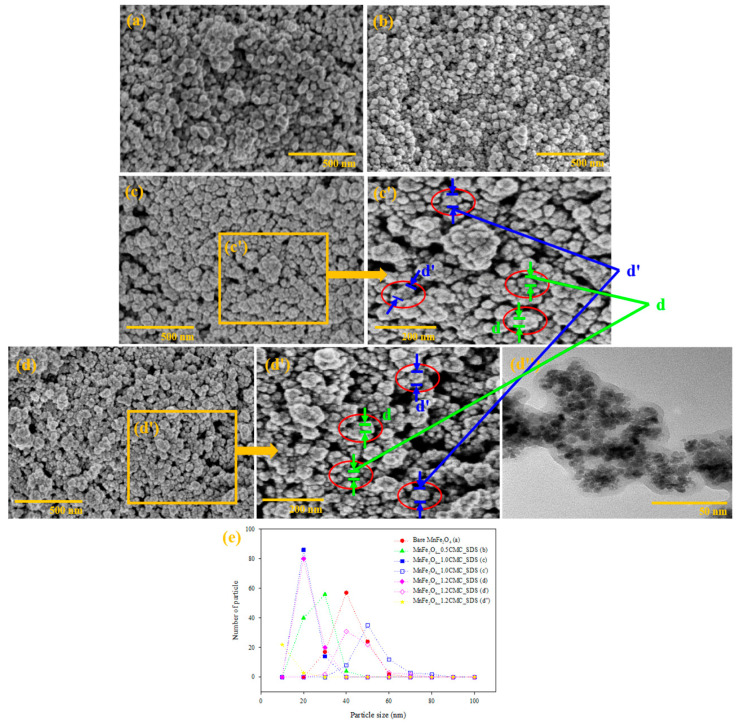
SEM and TEM images of the synthesized MnFe_2_O_4_ ferrite nanoparticles at various SDS surfactant concentrations: (**a**) SEM image of bare MnFe_2_O_4_, (**b**) SEM image of MnFe_2_O_4__0.5CMC_SDS, (**c**) SEM image of MnFe_2_O_4__1.0CMC_SDS, (**c’**) inset SEM image of (**c**) MnFe_2_O_4__1.0CMC_SDS, (**d**) SEM image of MnFe_2_O_4__1.2CMC_SDS, (**d’**) inset SEM image of (**d**) MnFe_2_O_4__1.2CMC_SDS, (**d″**) TEM image of MnFe_2_O_4__1.2CMC_SDS, and (**e**) the particle size distributions.

**Figure 6:**
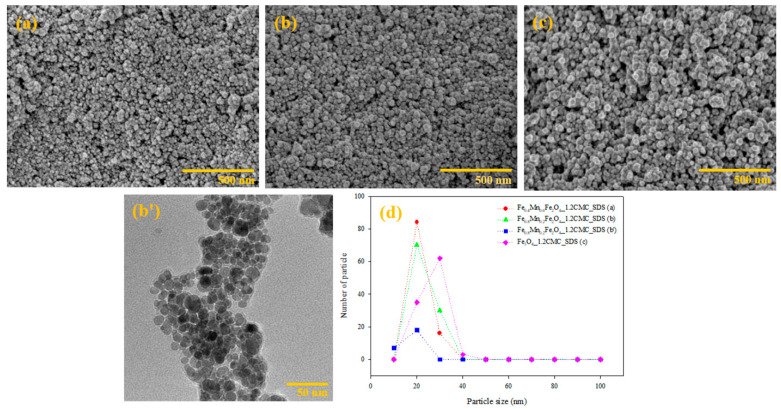
SEM and TEM images of the synthesized Fe_(1−x)_Mn_x_Fe_2_O_4__1.2CMC_SDS ferrite nanoparticles: (**a**) SEM image of Fe_0.4_Mn_0.6_Fe_2_O_4__1.2CMC_SDS, (**b**) SEM image of Fe_0.8_Mn_0.2_Fe_2_O_4__1.2CMC_SDS, (**b’**) TEM image of Fe_0.8_Mn_0.2_Fe_2_O_4__1.2CMC_SDS, (**c**) SEM image of Fe_3_O_4__1.2CMC_SDS, and (**d**) the particle size distributions.

The morphological characteristics namely the shape, size, and degree of aggregation of the MnFe_2_O_4_ and Fe_(1−x)_Mn_x_Fe_2_O_4__1.2CMC_SDS of various synthesized conditions were examined by the SEM and TEM techniques, as shown in Figure 5a–d″ or Figure 6a–c.

From the SEM images, all ferrite nanoparticles are of spherical shapes with the apparent individual particle sizes of 36 ± 6 nm (bare MnFe_2_O_4_), 22 ± 5 nm (MnFe_2_O_4__0.5CMC_SDS), 17 ± 3 nm (MnFe_2_O_4__1.0CMC_SDS), and 17 ± 3 nm (MnFe_2_O_4__1.2CMC_SDS), as tabulated in Table 1. The individual particle size decreases with increasing SDS surfactant concentration, consistent with the decreasing crystallite size in Table 1. Adding the SDS surfactant reduces the crystallite and particle sizes as the SDS micelles disrupt the particle–particle interaction and the crystal growth rate [21,30,44,49]. For the MnFe_2_O_4__1.0CMC_SDS and Fe_(1−x)_Mn_x_Fe_2_O_4__1.2CMC_SDS, the aggregated particle sizes (d’) are 49 ± 8 nm and 40 ± 8 nm, respectively. The particle aggregation possibly occurred from the removal of SDS after the synthesis to form the larger particle sizes.

For the effect of increasing Fe^2+^ ions substitution using SDS at 1.2 CMC, the apparent individual particle sizes of the MnFe_2_O_4__1.2CMC_SDS, Fe_0.4_Mn_0.6_Fe_2_O_4__1.2CMC_SDS, Fe_0.8_Mn_0.2_Fe_2_O_4__1.2CMC_SDS, and Fe_3_O_4__1.2CMC_SDS are 17 ± 3, 18 ± 2, 18 ± 3, and 22 ± 4 nm, respectively, as tabulated in Table 1. The individual particle size increases slightly with increasing the Fe^2+^ ions substitution, possibly due to the relatively higher crystal growth rates, consistent with the increase in crystallite size as shown in Table 1 [46,52]. 

The TEM images of the MnFe_2_O_4__1.2CMC_SDS and Fe_0.8_Mn_0.2_Fe_2_O_4__1.2CMC_SDS are shown in Figure 5d″ or Figure 6b’, respectively. The individual spherical particle sizes of MnFe_2_O_4__1.2CMC_SDS and Fe_0.8_Mn_0.2_Fe_2_O_4__1.2CMC_SDS are 8 ± 2 and 11 ± 2 nm, respectively. Thus, the TEM images indicate that the particle size also increases with increasing Fe^2+^ ions substituted consistent with the SEM and XRD results.

### 3.2. Electrical Conductivity Analysis

Generally, the spinel ferrites are classified as semiconductor materials in which the electrical conduction takes place by the electron exchange between the same ion elements. For the MnFe_2_O_4_ ferrite nanoparticles, it has two electrical conduction hopping processes: (i) the hole hopping between Mn^2+^ and Mn^3+^ ions; and (ii) the electron hopping between Fe^2+^ and Fe^3+^ ions [14,53,54,55,56]. The electrical conductivity of MnFe_2_O_4__1.2CMC_SDS, Fe_0.4_Mn_0.6_Fe_2_O_4__1.2CMC_SDS, Fe_0.8_Mn_0.2_Fe_2_O_4__1.2CMC_SDS, and Fe_3_O_4__1.2CMC_SDS are 2.04 × 10^−3^, 3.26 × 10^−3^, 4.08 × 10^−3^, and 9.68 × 10^−3^ S/cm, respectively, as shown in Figure S2. The MnFe_2_O_4__1.2CMC_SDS has a lower electrical conductivity than others, simply because it has no Mn^3+^ and Fe^2+^ ions in the ferrite structure; it has only the hopping process between Mn^2+^ and Fe^3+^ ions. The electrical conductivity increases with increasing the Fe^2+^ substitution where the Fe_3_O_4_ attains the highest electrical conductivity. The increase in the number of Fe^2+^ substituted allows the Fe^3+^ ions to remain at the octahedral site (B site). Therefore, the electron hopping between Fe^2+^ and Fe^3+^ ions is promoted, resulting in the higher electrical conductivity; a high electrical conductivity provides a potential for uses in actuator, bioengineering, and biomedical applications, particularly the actuations under an applied electric field, as shown in previous publications. Petcharoen et al. studied the actuation under applied electric and electromagnetic field of magnetite nanoparticles (Fe_3_O_4_)/polyurethane (PU) composites; the storage modulus (G’) and the storage modulus sensitivity (ΔG’/G’_0_) of the composites increased with increasing Fe_3_O_4_ contents under applied electric field, and the deflection responses also increased with increasing Fe_3_O_4_ contents under applied electric and electromagnetic fields [13]. Mohanty et al. developed the polyvinyl alcohol (PVA)/magnetite (Fe_3_O_4_) nanocomposite membrane; the increase of Fe_3_O_4_ contents increased the electrical conductivity, resulting in the increase in charge transport of the nanocomposite membrane under applied electric field [57].

### 3.3. Vibrating Sample Magnetometer (VSM) Analysis

**Figure 7:**
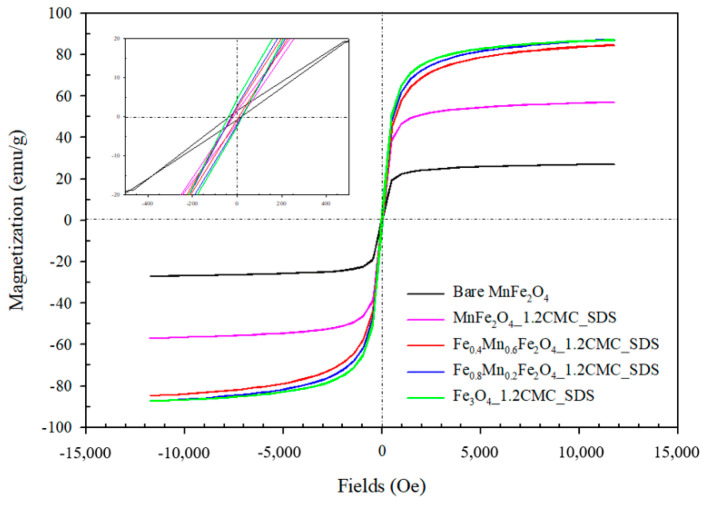
Magnetization of the synthesized bare MnFe_2_O_4_ and Fe_(1-x)_Mn_x_Fe_2_O_4__1.2CMC_SDS ferrite nanoparticles.

The magnetic properties of magnetic materials are known to depend on the structure, size, shape, and composition [58]. The magnetization versus magnetic field plots (M-H hysteresis loop) provide the magnetism behavior as well as the values of saturation magnetization (M_s_), coercivity (H_c_), and remanent magnetization (M_r_). The magnetic properties of the bare MnFe_2_O_4_ and Fe_(1-x)_Mn_x_Fe_2_O_4__1.2CMC_SDS are shown in Figure 7 and tabulated in Table 2. All hysteresis loops of the ferrite nanoparticles are quite narrow with small amounts of energy dissipated, thus exhibiting the soft ferromagnetic behavior at room temperature, as also suggested by the inset plots of Figure 7, Figures S3 and S4 [21,58,59]. Moreover, the small H_c_ could imply that the energy barriers are larger than the thermal, energy and hence the magnetic moments will be blocked in the parallel or anti-parallel directions, resulting in the small hysteresis loop (not superparamagnetic). When the energy barriers are smaller than the thermal energy, the energy barriers are overcome and the rotation of the magnetic moments becomes spontaneous under applied the magnetic field, resulting in the non-hysteresis loop (superparamagnetic).

#### 3.3.1. Effect of Surfactant Types

The effect of surfactant types, namely CTAB, SDS, and Tween20, on the magnetic properties are shown in Figure S3. For the MnFe_2_O_4_ ferrite nanoparticle with SDS, the M_s_ value is 73 emu/g, relatively higher than CTAB (67 emu/g) and Tween20 (57 emu/g). This is because only the SDS molecules can provide the steric repulsive force to balance the magnetic and van der Waals attractive forces between the nanoparticles [44,60]. All MnFe_2_O_4_ with surfactants possesses higher M_s_ values than the bare MnFe_2_O_4_ (27 emu/g), as the surfactants can influence the crystallite and particle sizes, the nanoparticle aggregation, and consequently the surface spin disorder [44,49,60]. The SDS surfactant appears to be the most effective surfactant to provide the high M_s_ value.

#### 3.3.2. Effect of SDS Surfactant Concentrations

The M_s_ values of the bare MnFe_2_O_4_ and the MnFe_2_O_4_ with various SDS concentrations are tabulated in Table 2 and Figure S4. The M_s_ value increases from 27 emu/g to 73 emu/g, with increasing SDS concentration from 0.0 to 1.0 CMC. At 1.2 CMC, the M_s_ value decreases to 57 emu/g. The corresponding individual particle sizes (d) are 36 nm, 22 nm, and the aggregated particle sizes (d’) are 49 nm, and 40 nm, with increasing SDS concentration, as tabulated in Table 1. The SDS presence affects the M_s_ value as the SDS molecules can influence the particle–particle interaction and consequently the surface spin disorder [30,49]. The individual particle size (d) here refers to the visibly apparent particle sizes in Figure 5a,b, and the aggregated particle sizes (d’) can be seen in Figure 5c’,d’; the latter particles consist of many smaller and aggregated particles. The M_s_ values in Table 2 can be correlated with the particle size (d) of bare MnFe_2_O_4_ and MnFe_2_O_4__0.5CMC_SDS and the aggregated particle sizes (d’) of MnFe_2_O_4__1.0CMC_SDS and MnFe_2_O_4__1.2CMC_SDS, as shown in Table 1. Here, M_s_ increases with particle size due to the reduction in spin disorders from the particle aggregation, after the removal of SDS [49].

The optimum surfactant concentrations to obtain the highest M_s_ values have been reported. Zhang et al. reported the M_s_ value of MnFe_2_O_4_ nanoparticles increased with increasing CTAB surfactant concentration as synthesized by a co-precipitation method; the highest M_s_ value was 54.5 emu/g at 0.5 M of CTAB surfactant; it became lower at higher concentrations [44]. Vadivel et al. reported that the M_s_ value of CoFe_2_O_4_ nanoparticles increased with increasing SDS surfactant concentration; the highest M_s_ value was 138.75 emu/g at 0.08 M of SDS surfactant [49]. The M_s_ values of the synthesized MnFe_2_O_4_ in this work are in the range of 15–69 emu/g, consistent with other previous reports [11,17,18,23,24,32,33,34,35,39,41,61]. When comparing the M_s_ values in the bulk or film forms, the M_s_ value of the presently synthesized MnFe_2_O_4_ nanoparticle is lower than that of the bulk form at 82 emu/g [61], but higher than the film form at 38 emu/g [61].

#### 3.3.3. Effect of Mn^2+^:Fe^2+^ Molar Ratio Substitutions

Under the effect of Fe^2+^ ion substitutions, the M_s_ value increases with increasing Fe^2+^ substituted into the MnFe_2_O_4_; the Fe_0.8_Mn_0.2_Fe_2_O_4_ and Fe_3_O_4_ attain the highest M_s_ values of 88 and 87 emu/g, respectively, as tabulated in Table 2. The results can be explained by three factors. (i) The first factor is the cation distributions of Fe^3+^, Mn^2+^, and Fe^2+^ substituted into the A and B sites. As the Fe^2+^ ions are substituted for the Mn^2+^ ions, the Fe^2+^ and Mn^2+^ ions preferably stay at the A site, while the Fe^3+^ ions prefer to stay at the B site [31,41,62,63]. (ii) The second factor is the crystallite and particle sizes: the substitution of Fe^2+^ ions into the MnFe_2_O_4_ slightly increases the crystallite and particle sizes (Table 1) along with the lower surface-to-volume ratio and the lower surface spin disorder [31,44,47]. Hence, the M_s_ value increases with increasing crystallite and particle sizes, as observed from the XRD and SEM results. (iii) The third factor is the spin–spin interaction; as more Fe^2+^ ions are added into the MnFe_2_O_4_, the M_s_ value also increases because of the stronger spin–spin interaction as related to the decrease in lattice constant as shown in Table 1. The spin–spin interactions determine the Curie temperature (T_c_) and hence the temperature variation of the magnetization, resulting in the different M_s_ values at room temperature. In short, the M_s_ value increases with increasing spin–spin interaction [63,64]. Moreover, the H_c_ values in Table 2 are close to zero, which suggests that the energy barriers are larger than the thermal energy, and the magnetic moments will be blocked in the parallel or anti-parallel directions, resulting in the small hysteresis loop and indicating the soft ferromagnetic behavior in all samples.

It may be noted that the M_s_ value of the synthesized Fe_0.8_Mn_0.2_Fe_2_O_4__1.2CMC_SDS (88 emu/g) is higher than those of other works. Modaresi et al. and Doaga et al. synthesized the Fe_0.8_Mn_0.2_Fe_2_O_4_ ferrite nanoparticles by using the co-precipitation method; the M_s_ values were 18.2 and 56.3 emu/g, respectively [31,39]. Guner et al. synthesized the Fe_0.8_Mn_0.2_Fe_2_O_4_ ferrite nanoparticles by using the polyol method; the M_s_ value was 48.32 emu/g [41].

The magnetic moments in the units of Bohr magneton of the synthesized ferrites were calculated by Equation (10) [41]:

Magnetic moments in the units of Bohr magneton = (M × σ_s_)/(N_A_ × µ_B_) (10)

where M is the molecular weight of each sample (g/mol), σ_s_ is the specific magnetization (emu/g) as evaluated from the specific magnetization as a function of 1/H^2^ plot, N_A_ is the Avogadro’s number (6.02 × 10^23^ mol^−1^), and µ_B_ is the Bohr magneton (9.27 × 10^−21^ Erg/Oe). All magnetic moments of the MnFe_2_O_4_ ferrite nanoparticles under various SDS surfactant concentrations and the Fe_(1−x)_Mn_x_Fe_2_O_4__1.2CMC_SDS ferrite nanoparticles under various Fe^2+^ ions substituted are tabulated in Table 2. The magnetic moment increases with increasing SDS concentration and Fe^2+^ substituted into the MnFe_2_O_4_ ferrite nanoparticles. A low magnetic moment occurs with a low M_s_ value resulting from a specifically preferred cation distribution of the metal ions in the A and B sites, and a surface spin disorder [31,39,41]. The theoretical magnetic moments of Mn^2+^, Fe^3+^, and Fe^2+^ ions are 5 µ_B_, 5 µ_B_, and 4 µ_B_, respectively, as derived from the numbers of unpaired electrons in the 3D orbitals [31].

### 3.4. Dispersion Property Fe_(1−x)_Mn_x_Fe_2_O_4_ Ferrite Nanoparticles

**Figure 8:**
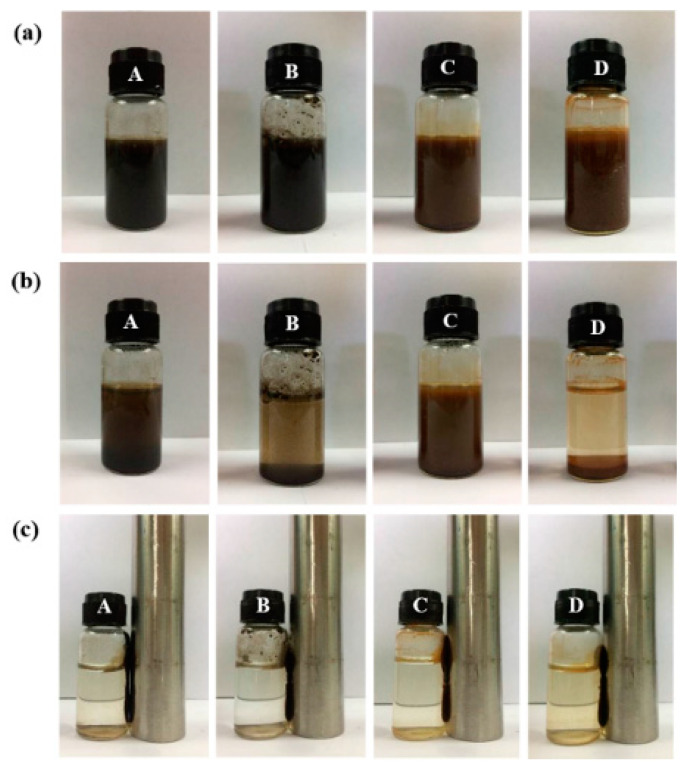
Dispersion property of the synthesized bare MnFe_2_O_4_ (A), MnFe_2_O_4_ _1.2CMC_SDS (B), Fe_0.8_Mn_0.2_Fe_2_O_4__1.2CMC_SDS (C), Fe_3_O_4__1.2CMC_SDS ferrite nanoparticles (D): (**a**) after ultrasonication; (**b**) 15 min; (**c**) under applied an external magnetic field.

The dispersion property in water of the synthesized bare MnFe_2_O_4_ and Fe_(1−x)_Mn_x_Fe_2_O_4__1.2CMC_SDS ferrite nanoparticles with various Fe^2+^ ions substituted was investigated next, as this property is an important feature in various applications such as magnetic fluid, catalysis, bio-sensing, magnetic resonance imaging (MRI), and hyperthermia treatment [65,66]. In a recent work, Mei et al. used sodium oleate (NaOL) to modify the surface of magnetite nanoparticles to improve the selective and functional water dispersion to enhance the efficiency of plasmonic bio-sensing [67]. In this work, all ferrite nanoparticles were dispersed in water by using the sonication bath for 15 min, and then the sedimentation of precipitate was observed during a period of 7 days. The results of the sedimentation experiment are shown in Figure 8. The Fe_0.8_Mn_0.2_Fe_2_O_4__1.2CMC_SDS ferrite nanoparticles (C) provided a longer-lasting dispersion in water than the bare MnFe_2_O_4_ (A), MnFe_2_O_4__1.2CMC_SDS (B), and Fe_3_O_4__1.2CMC_SDS ferrite nanoparticles (D). Generally, the dispersion is promoted by the Brownian motion of the nanoparticles [68]. On the other hand, the rapidly precipitated magnetic nanoparticles are due to the strong magnetic dipole–dipole interaction between nanoparticles and the gravity force [29,65]. 

**Figure 9:**
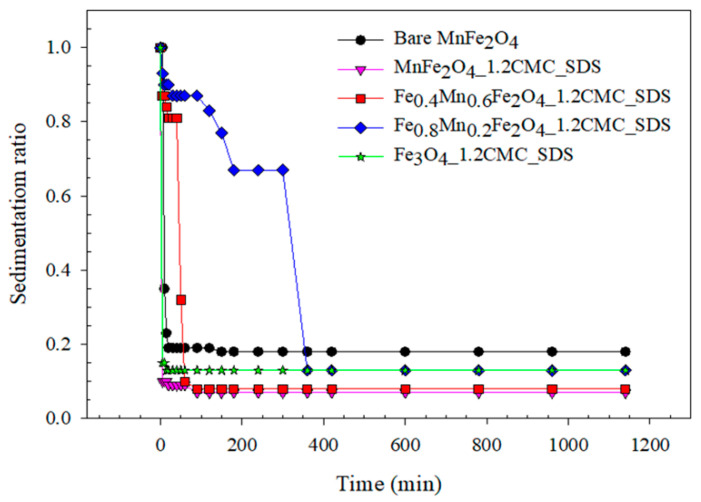
Sedimentation ratio as a function of time of the synthesized bare MnFe_2_O_4_ and Fe_(1−x)_Mn_x_Fe_2_O_4__1.2CMC_SDS ferrite nanoparticles.

All ferrite nanoparticles can be separated from the water by using an external magnetic field. The sedimentation ratios of the bare MnFe_2_O_4_ and Fe_(1−x_)Mn_x_Fe_2_O_4__1.2CMC_SDS ferrite nanoparticles were calculated from the heights of the precipitates divided by the initial heights as a function of time, as shown in Figure 9. The results support the dispersion stability results of magnetic nanoparticles in Figure 8. The dispersions of the bare MnFe_2_O_4_, MnFe_2_O_4__1.2CMC_SDS, and Fe_3_O_4__1.2CMC_SDS ferrite nanoparticles are relatively poor as the sedimentation ratios drop close to zero within a short time, whereas the Fe_0.8_Mn_0.2_Fe_2_O_4__1.2CMC_SDS ferrite nanoparticle can be dispersed in water for a period of 360 min. Surfactants, polymeric compounds, organic materials, or inorganic materials have been commonly used to improve the dispersion stability of magnetic materials [67,68]. Petcharoen et al. used two fatty acids (oleic acid (OA) and hexanoic acid (HA)) as coating agents for the synthesized magnetite nanoparticles; they illustrated that the oleic acid-coated magnetite nanoparticle was more stable for a longer period of time in the water than the hexanoic acid and bare samples [43].

## 4. Conclusions

The MnFe_2_O_4_ ferrite nanoparticles were successfully synthesized by the surfactant-assisted co-precipitation under the effects of surfactant types, surfactant concentrations, and Mn^2+^:Fe^2+^ molar ratio substitutions. The presence of SDS generally reduced the crystallite and individual particle sizes whereas the increase of Fe^2+^ ions substituted increased the crystallite and particle sizes due to the increased crystal growth rate. Adding Fe^2+^ ions changed the crystal structure from the mixed spinel (MnFe_2_O_4_) to the inverse spinel (Fe_3_O_4_). All of the ferrite nanoparticles showed a spherical nanoparticle shape, with individual sizes between 17–22 nm depending on the synthesis conditions. The electrical conductivity increased by increasing the number of Fe^2+^ ions substituted. The highest electrical conductivity of 9.68 × 10^−3^ S/cm was obtained from the Fe_3_O_4_ ferrite nanoparticle. For the magnetic properties, all ferrite nanoparticles demonstrated the soft ferromagnetic behavior. The highest M_s_ value of 88 emu/g was obtained from the Fe_0.8_Mn_0.2_Fe_2_O_4__1.2CMC_SDS, with the nanoparticle size of 18 ± 3 nm. The Fe_0.8_Mn_0.2_Fe_2_O_4__1.2CMC_SDS ferrite nanoparticle took the longest time to sediment in water. The present results suggest that the SDS surfactant was influential as the template to control the crystallite and particle sizes to improve the magnetic properties, and the Fe^2+^ ions substitution was shown to be a significant route to improve the electrical and magnetic properties. The synthesized nano-magnetic particles have the potential to be used [7,13,69,70,71,72,73,74,75,76,77,78,79]: in soft ferrogels and ferro-elastomers for the magnetic-deformation or electro-magnetic-deformation in actuator applications; in the drug-loaded magnetic nanocomposites for controlling the amount of drug release in drug release systems; in magnetic nanocarriers or magnetic nanocomposites for killing cancer cells by thermotherapy via ultrasounds, microwaves, or radiofrequency radiation in hyperthermia applications, based on natural and synthetic polymers.

## Figures and Tables

**Table 1:** Average crystallite size (D), lattice constant (a), unit volume cell (V_cell_), hopping length for tetrahedral site (L_A_), hopping length for octahedral site (L_B_), X-ray density ($\rho_{x-\mathrm{ray}}$ ), specific surface area (S), particle size (d), aggregated particle size (d’) of the synthesized bare MnFe_2_O_4_, MnFe_2_O_4_ at various SDS surfactant concentrations, and Fe_(1−x)_Mn_x_Fe_2_O_4__1.2CMC_SDS ferrite nanoparticles.

Sample	D (nm)	a (Å)	V_cell_ (Å^3^)	L_A_ (Å)	L_B_ (Å)	$\rho_{\mathrm{X-ray}}$ (×10^3^ Kg/m^3^)	S (m^2^/g)	d (nm)	d’ (nm)
Bare MnFe_2_O_4_	10.6	8.39	591	3.66	2.99	5.19	111	36 ± 6	-
MnFe_2_O_4__0.5CMC_SDS	10.5	8.39	591	3.66	2.99	5.19	113	22 ± 5	-
MnFe_2_O_4__1.0CMC_SDS	8.47	8.39	591	3.63	2.97	5.19	137	17 ± 3	49 ± 8
MnFe_2_O_4__1.2CMC_SDS	7.80	8.46	604	3.66	2.99	5.07	152	17 ± 3	40 ± 8
Fe_0.4_Mn_0.6_Fe_2_O_4__1.2CMC_SDS	7.82	8.39	590	3.63	2.97	5.20	148	18 ± 2	-
Fe_0.8_Mn_0.2_Fe_2_O_4__1.2CMC_SDS	10.8	8.35	582	3.62	2.95	5.28	106	18 ± 3	-
Fe_3_O_4__1.2CMC_SDS	13.3	8.30	572	3.60	2.94	5.38	83.9	22 ± 4	-

**Table 2:** The saturation magnetization (M_s_), coercivity (H_c_), remanent magnetization (M_r_), and magnetic moment in the units of Bohr magneton of the synthesized bare MnFe_2_O_4_ and MnFe_2_O_4_ at various SDS surfactant concentrations, and Fe_(1−x)_Mn_x_Fe_2_O_4__1.2CMC_SDS ferrite nanoparticles.

Sample	M_s_ (emu/g)	H_c_ (Oe)	M_r_ (emu/g)	Magnetic Moment in the Units of Bohr Magneton
Bare MnFe_2_O_4_	27	27	1.6	1.1
MnFe_2_O_4__0.5CMC_SDS	55-6	19	2.4	2.3
MnFe_2_O_4__1.0CMC_SDS	73	20	2.8	3.1
MnFe_2_O_4__1.2CMC_SDS	57	17	1.5	2.4
Fe_0.4_Mn_0.6_Fe_2_O_4__1.2CMC_SDS	85	16	2.4	3.6
Fe_0.8_Mn_0.2_Fe_2_O_4__1.2CMC_SDS	88	23	2.8	3.7
Fe_3_O_4__1.2CMC_SDS	87	31	4.6	3.7

## Data Availability

The data presented in this study are available on request from the corresponding author. The data are not publicly available due to the author’s readiness to provide it on request.

## Supplementary Materials

The following are available online at https://www.mdpi.com/article/10.3390/nano11040876/s1: Table S1: Average crystalline sizes (D) calculated by using the Debye–Scherrer’s Equation and Williamson–Hall Plot, and ε values from Williamson–Hall Plot of the synthesized Fe_(1-x)_Mn_x_Fe_2_O_4__1.2CMC_SDS ferrite nanoparticles’; Figure S1.1: Williamson–Hall plot of MnFe_2_O_4__1.2CMC_SDS ferrite nanoparticles; Figure S1.2: Williamson–Hall plot of Fe_0.4_Mn_0.6_Fe_2_O_4__1.2CMC_SDS ferrite nanoparticles; Figure S1.3: Williamson–Hall plot of Fe_0.8_Mn_0.2_Fe_2_O_4__1.2CMC_SDS ferrite nanoparticles; Figure S1.4: Williamson–Hall plot of Fe_3_O_4__1.2CMC_SDS ferrite nanoparticles; Figure S2: Electrical conductivity of the synthesized bare MnFe_2_O_4_ and Fe_(1−x)_Mn_x_Fe_2_O_4__1.2CMC_SDS ferrite nanoparticles; Figure S3: Magnetizations of the synthesized MnFe_2_O_4_ ferrite nanoparticles under various surfactant types; and Figure S4: Magnetizations of the synthesized bare MnFe_2_O_4_ and MnFe_2_O_4_ ferrite nanoparticles under various SDS surfactant concentrations.

## Abbreviations

Manganese ferrite, MnFe_2_O_4_; sodium dodecyl sulfate, SDS; iron (II), Fe^2+^; manganese substituted with iron (II) ferrite, Fe_(1-x)_Mn_x_Fe_2_O_4_; magnetite ferrite, Fe_3_O_4_; cobalt ferrite, CoFe_2_O_4_; cetyltrimethylammonium bromide, CTAB; iron (III) chloride, FeCl_3_∙2H_2_O; manganese (II) chloride, MnCl_2_; iron (II) sulphate, FeSO_4_∙7H_2_O; polysorbate20, Tween20; 25 wt% Ammonium hydroxide solution, NH_4_OH; critical micelle concentrations of surfactant, CMC; saturation magnetization, M_s_; coercivity, H_c_; remanent magnetization, M_r_; specific conductivity, σ; specific resistivity, ρ; sheet resistivity, Rs; measured current, I; geometric correction factor, K; applied voltage, V; disc thickness, t; average crystallite size, D; shape factor that depends on the structure of the crystallite, K; X-ray wavelength, λ; full width at half-maximum (FWHM), β; Bragg’s angle, θ; lattice constant, a; interplane distance, d; volume of unit cell, V_cell_; hopping length for tetrahedral site, L_A_; hopping length for octahedral site, L_B_; x-ray density, ρ_x-ray_; molecular weight, M; Avogadro’s number, N_A_; specific surface area, S; aggregated particle size, d’; particle size, d; storage modulus, G’; storage modulus sensitivity, ΔG’/G’_0_; polyurethane, PU; polyvinyl alcohol, PVA; curie temperature, T_c_; specific magnetization, σ_s_; bohr magneton, µ_B_; magnetic resonance imaging, MRI; sodium oleate, NaOL; oleic acid, OA; hexanoic acid, HA; fourier transformed infrared spectrometer, FT-IR; thermogravimetric analyzer, TGA; wide angle X-ray diffractometer, XRD; high-resolution field-emission scanning electron microscope with the energy disper-sive X-ray, FE-SEM-EDX; x-ray photoelectron spectroscopy, XPS; high-resolution field-emission scanning electron microscope, FE-SEM; transmission electron microscope, TEM; vibrating sample magnetometer, VSM; derivative thermograms, DTG.
