# Supplementary material for: Synthesis and Characterization of Fe0.8Mn0.2Fe2O4 Ferrite Nanoparticle with High Saturation Magnetization via the Surfactant Assisted Co-Precipitation
Source: Nanomaterials (Basel). 2021 Mar 30;11(4):876. doi: 10.3390/nano11040876 (PMC8067334; doi:10.3390/nano11040876)
Supplement: Supplementary file 1 [file nanomaterials-11-00876-s001.pdf]

nanomaterials

Supplementary Materials

Synthesis and Characterization of $Fe_{0.8}Mn_{0.2}Fe_2O_4$ Ferrite Nanoparticle with High Saturation Magnetization via the Surfactant Assisted Co-Precipitation

Kornkanok Rotjanasuworapong [1], Wanchai Lerdwijitjarud [2] and Anuvat Sirivat [1,*]

1 Conductive and Electroactive Polymers Research Unit, The Petroleum and Petrochemical College, Chulalongkorn University, Bangkok 10330, Thailand; kornkanok.rotj@gmail.com
2 Department of Materials Science and Engineering, Faculty of Engineering and Industrial Technology, Silpakorn University, Nakorn Pathom 73000, Thailand; lerdwijitjarud_w@su.ac.th
* Correspondence: anuvat.s@chula.ac.th; Tel.: 662 218 4131

Table 1. Average crystalline sizes (D) calculated by using the Debye-Scherrer's Equation and Williamson-Hall Plot, and ε values from Williamson-Hall Plot of the synthesized $Fe_{(1-x)}Mn_xFe_2O_4$_1.2CMC_SDS ferrite nanoparticles.

Sample	D (nm)		ε value from Williamson-Hall Plot
	Debye-Scherrer's Equation	Williamson-Hall Plot	
$MnFe_2O_4$_1.2CMC_SDS	7.80	11.9	3.24×10^{-3}
$Fe_{0.4}Mn_{0.6}Fe_2O_4$_1.2CMC_SDS	7.82	12.8	4.01×10^{-3}
$Fe_{0.8}Mn_{0.2}Fe_2O_4$_1.2CMC_SDS	10.8	13.5	1.17×10^{-3}
Fe_3O_4_1.2CMC_SDS	13.3	14.0	7.21×10^{-5}

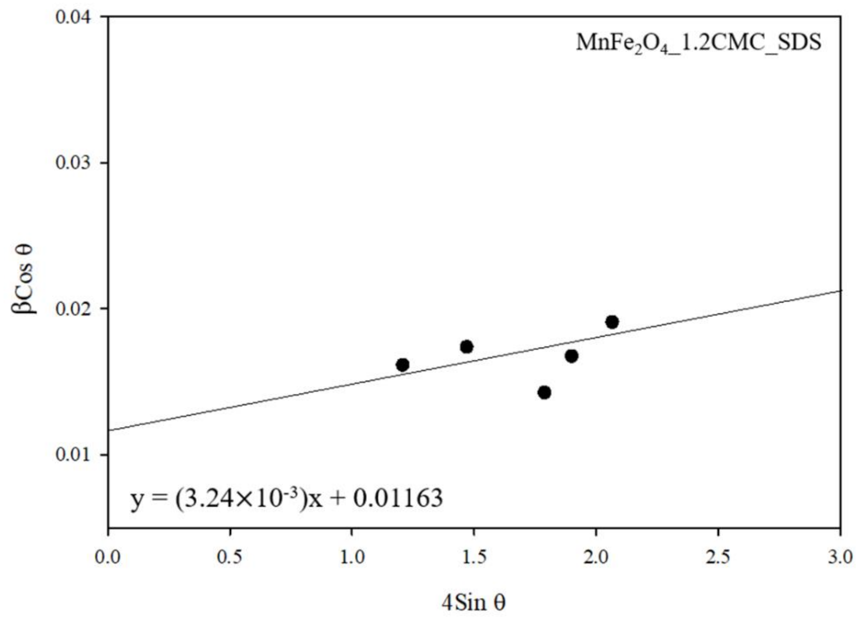

Figure S1. 1 Williamson-Hall plot of $MnFe_2O_4$_1.2CMC_SDS ferrite nanoparticles.

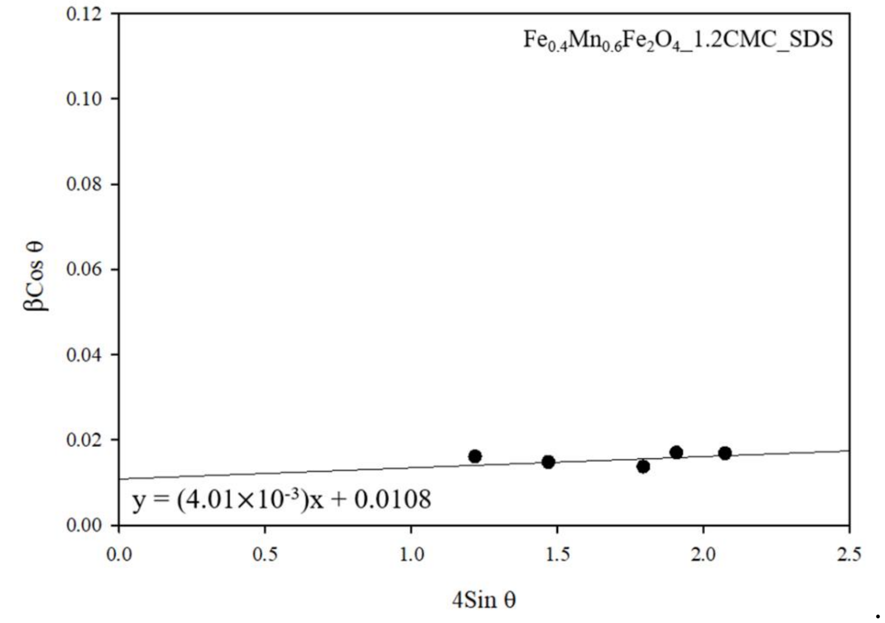

Figure S1. 2 Williamson-Hall plot of $Fe_{0.4}Mn_{0.6}Fe_2O_4$_1.2CMC_SDS ferrite nanoparticles.

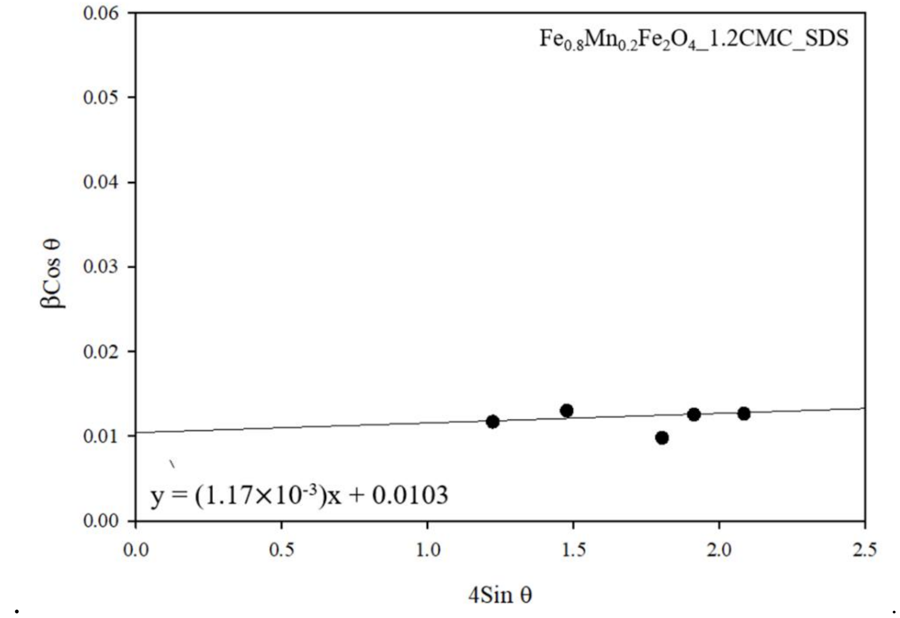

Figure 1. 3 Williamson-Hall plot of $Fe_{0.8}Mn_{0.2}Fe_2O_4$_1.2CMC_SDS ferrite nanoparticles.

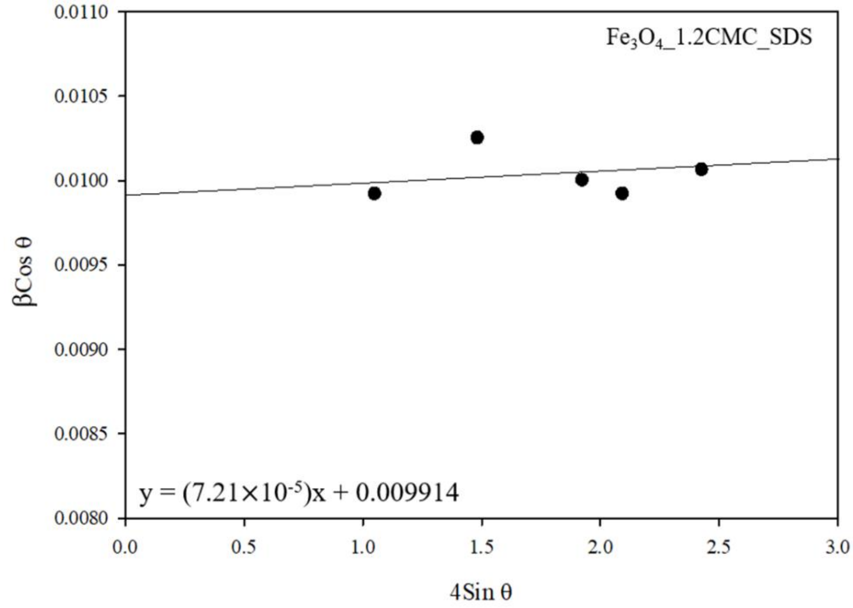

.

Figure 1. 4 Williamson-Hall plot of Fe_3O_4_1.2CMC_SDS ferrite nanoparticles.

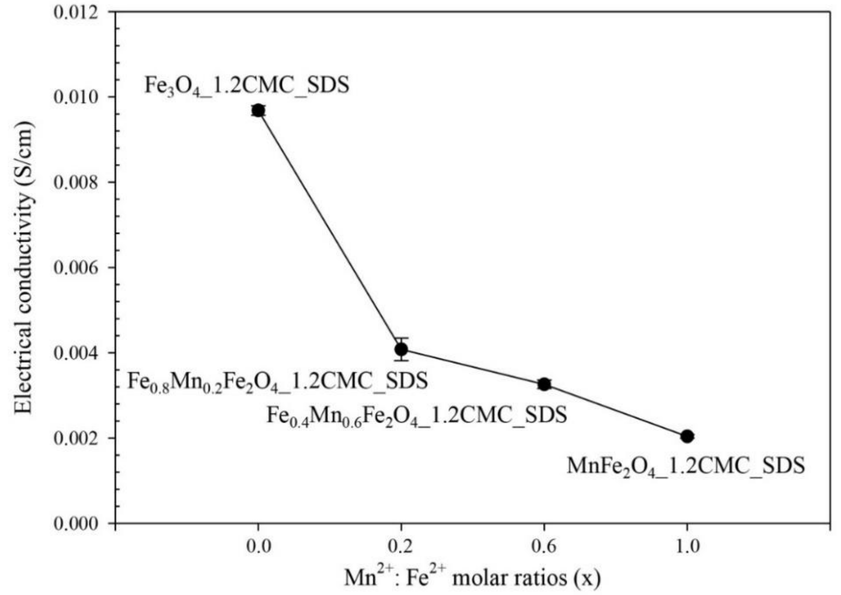

.

Figure S2. Electrical conductivity of the synthesized bare $MnFe_2O_4$ and $Fe_{(1-x)}Mn_xFe_2O_4$_1.2CMC_SDS ferrite nanoparticles.

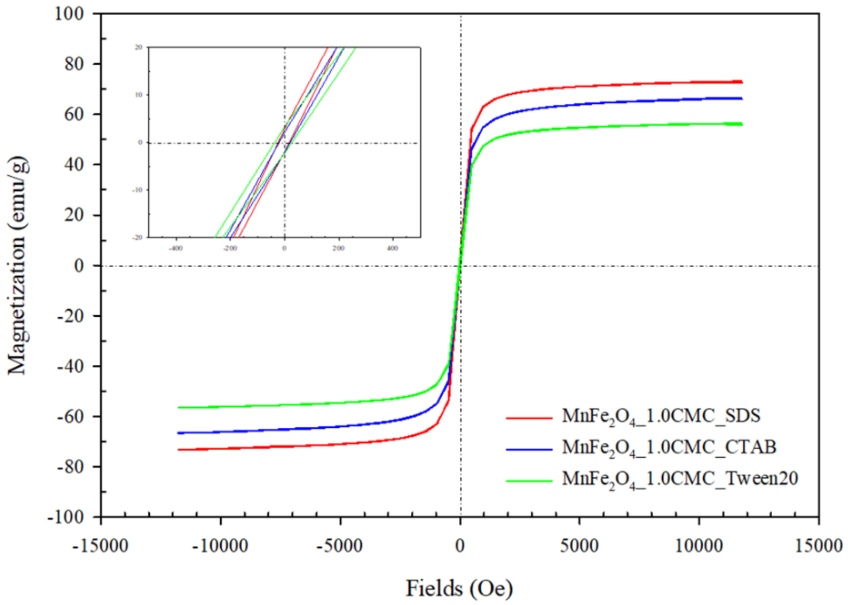

Figure S3. Magnetizations of the synthesized $MnFe_2O_4$ ferrite nanoparticles under various surfactant types.

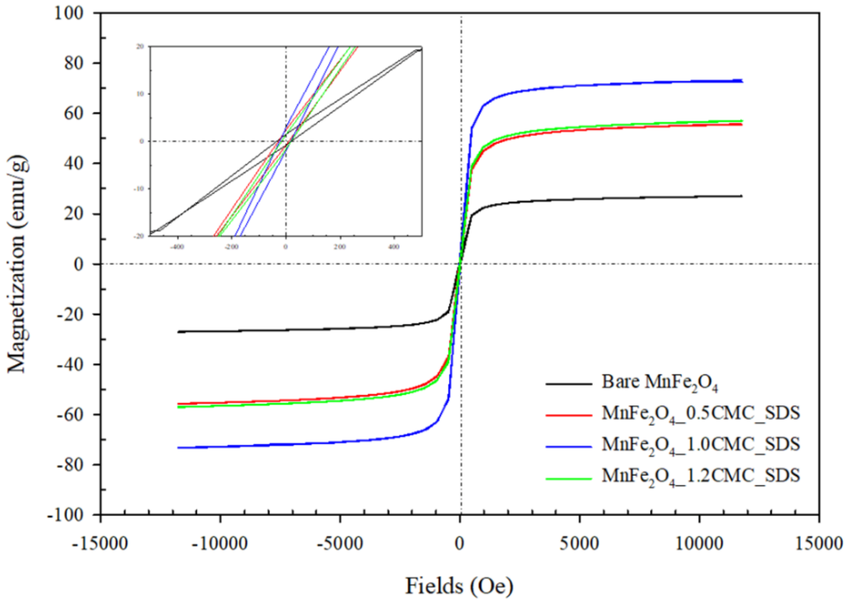

Figure 4. Magnetizations of the synthesized bare $MnFe_2O_4$ and $MnFe_2O_4$ ferrite nanoparticles under various SDS surfactant concentrations.
